# Effect of tumor necrosis factor inhibition on spinal inflammation and spinal ankylosis in SKG mice

**DOI:** 10.1038/s41598-019-54549-5

**Published:** 2019-11-29

**Authors:** Doo-Ho Lim, Eun-Ju Lee, Oh Chan Kwon, Seokchan Hong, Chang-Keun Lee, Bin Yoo, Jeehee Youn, Tae-Hwan Kim, Yong-Gil Kim

**Affiliations:** 1 Division of Rheumatology, Department of Internal Medicine, Ulsan University Hospital, University of Ulsan College of Medicine, Ulsan, Republic of Korea; 2 Asan Institute for Life Science, Asan Medical Center, Seoul, Republic of Korea; 3 Division of Rheumatology, Department of Internal Medicine, Yonsei University College of Medicine, Seoul, Republic of Korea; 4 Division of Rheumatology, Department of Internal Medicine, Asan Medical Center, University of Ulsan College of Medicine, Seoul, Republic of Korea; 5 Department of Anatomy and Cell Biology, College of Medicine, Hanyang University, Seoul, Republic of Korea; 6 Department of Rheumatology, Hanyang University Hospital for Rheumatic Diseases, Seoul, Republic of Korea

**Keywords:** Autoimmune diseases, Ankylosing spondylitis

## Abstract

To prevent spinal progression in ankylosing spondylitis, initiating TNF-inhibitor treatment as early as possible is suggested. However, the outcomes are inconsistent in previous clinical studies. Here, we investigated the effect of TNF inhibition alone on spinal progression when used during arthritis development in a murine model. We injected 8-week-old SKG mice with curdlan (curdlan group). We injected adalimumab at 3 and 9 weeks after the first curdlan injection (ADA group). The clinical scores of peripheral arthritis decreased in the ADA group at 3 weeks after first adalimumab injection. Using positron emission tomography–magnetic resonance imaging and histologic examination, spinal inflammation was observed in the curdlan group, and was significantly deceased in the ADA group. However, spinal osteoblast activities by imaging using OsteoSense 680 EX and bone metabolism-related cytokines such as receptor activator of nuclear factor-kappa B ligand, osteoprotegerin, Dickkopf-1, and sclerostin levels except IL-17A level were not different between the two groups. We conclude that treating TNF inhibitor alone reduced peripheral arthritis score and spinal inflammation in curdlan-injected SKG mice but did not decrease the spinal osteoblast activity, suggesting little effect on spinal ankylosis.

## Introduction

Ankylosing spondylitis (AS) is chronic inflammatory form of arthritis in which the spine and sacroiliac joint are primarily affected. AS is a prototype of spondyloarthritis (SpA) and is characterized by syndesmophytes and the ankylosis of the axial skeleton^1^. Excessive bone formation is an important factor in disease prognosis because it can impair the mobility of the spine. This consequently disturbs daily activity and reduces the quality of life of patients^2^. The exact pathologic process of bone formation in AS is still unclear; however, spinal inflammation may be associated with uncontrolled osteoproliferation that often results in the fusion of the affected joints^3^.

Tumor necrosis factor-alpha (TNF-α) plays an important role in the inflammatory response in AS. Moreover, TNF-α levels increase in the synovium and serum of AS patients^4^. TNF-α inhibitor treatment clearly improves the functional outcomes, disease activity, and the quality of life of patients who are unresponsive to conventional therapy^1^. However, previous studies have reported inconsistent findings regarding the effect of TNF-α inhibitor treatment on spinal radiographic progression in patients with AS^5–9^, whereas TNF-α inhibitors have been effective in preventing structural damage as well as reducing disease activity in psoriatic arthritis and rheumatoid arthritis (RA), in which bone erosion dominates bone formation^10,11^.

The roles of TNF-α in bone formation and inflammation in AS are yet to be fully elucidated. Previous *in vivo* studies demonstrated the uncoupling of bone formation and inflammation in the spine^12,13^ and showed that bone formation was controlled by the bone morphogenetic proteins, transforming growth factors, and Wnt proteins^14,15^. Meanwhile, inflammation is suggested to trigger the initiation of syndesmophytes by an inappropriate repair of inflammatory stress in patients with AS^16^ as recent clinical studies showed that TNF inhibitors decelerated spinal radiographic progression, especially in patients with early AS without syndesmophytes^17–21^. However, the effects of TNF inhibitors alone were difficult to verify clearly because standard treatments including nonsteroidal anti-inflammatory drugs were allowed in the clinical studies.

SKG mice develop chronic autoimmune inflammatory arthritis following systemic exposure to ß-glucan^22^. SKG mice harbor a genetic mutation in the SH2 domain of ZAP-70, which is a key signal transduction molecule in T cells^23,24^; as a result, SKG mice have an excess of arthritogenic T cells^22^. This results in chronic arthritis and extra-articular manifestations. Although SKG mice were initially used as a model of RA, Ruutu *et al*. reported that after systemically injecting curdlan, SKG mice developed the clinical characteristics of SpA, including sacroiliac joint arthritis, vertebral inflammation, enthesitis, peripheral arthritis, uveitis, and bowel inflammation^25^. Therefore, SKG mice can serve as an established animal model for examining the therapeutic effects of TNF inhibition on spinal inflammation.

In this study, we aimed to investigate the effect of a TNF inhibitor administered at the time of peripheral arthritis development on subsequent spinal inflammation and bone formation in an SKG mouse model.

## Results

### SKG mice developed spondyloarthritis phenotypes

**Figure 1:**
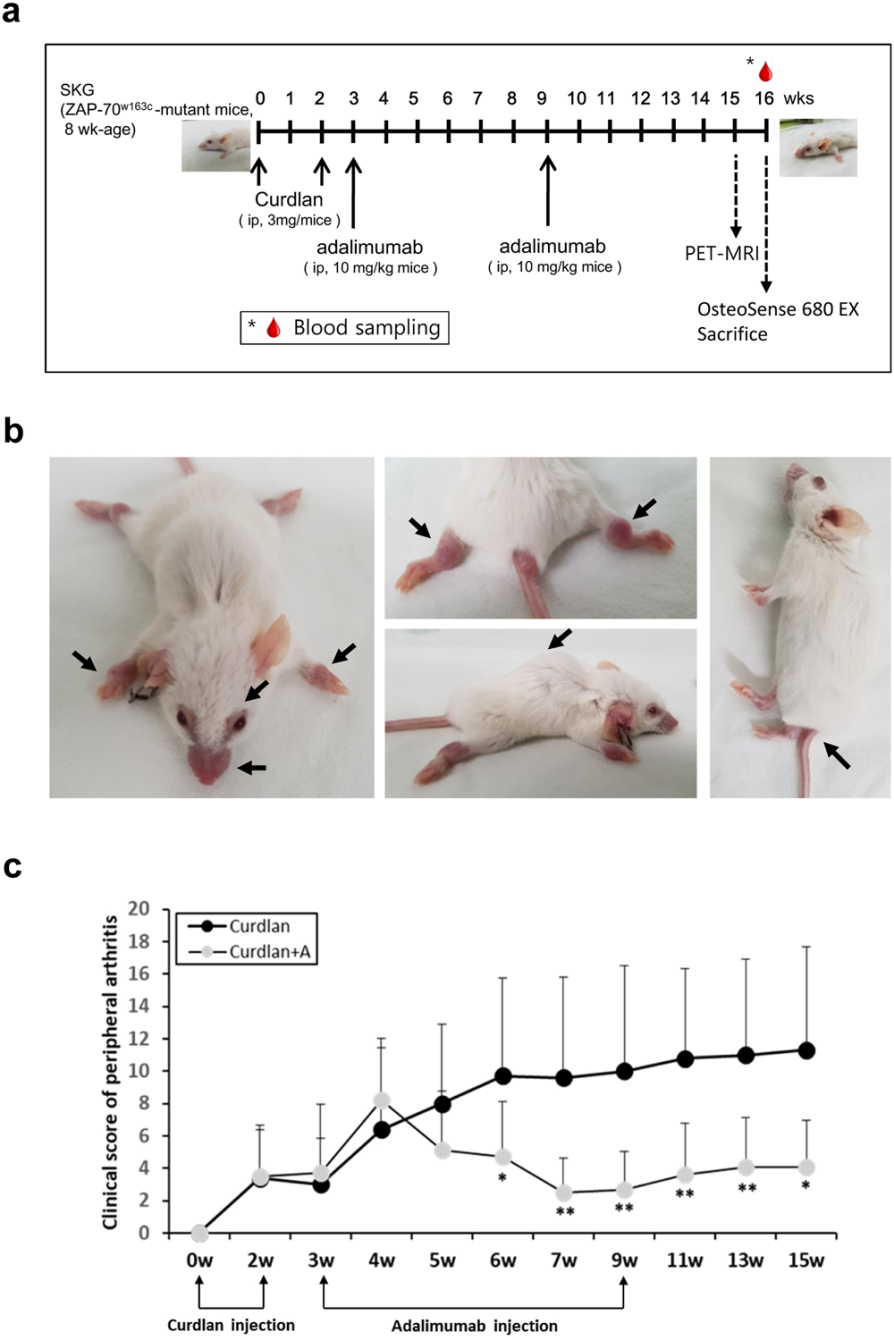
Experimental design and phenotype in curdlan-injected SKG mice. (**a**) Experimental design. (**b**) Peripheral arthritis and hair loss around the eye and nose were observed within 2 weeks after injecting curdlan, while tail and spinal deformities were examined 10 weeks after injecting curdlan in SKG mice. (**c**) Clinical score of peripheral arthritis. Error bars in A indicate mean ± SEM; *p < 0.05, **p < 0.01. Abbreviations: ip, intraperitoneal injection; curdlan + A, curdlan + adalimumab.

As shown in Fig. 1b, all SKG mice developed inflammatory arthritis in peripheral joints with soft tissue swelling within 2–3 weeks after the first intraperitoneal injection of 3-mg curdlan, whereas phosphate-buffered saline (PBS)-injected SKG mice did not show any features of inflammation. At 10 weeks after injecting curdlan, redness, swelling, and deformity along the tail and the hunching of the upper body were observed (Fig. 1b).

The effect of adalimumab on peripheral arthritis in curdlan-injected SKG mice was examined. Weekly observations showed that the clinical scores of peripheral arthritis markedly improved in adalimumab-treated mice, beginning at 2 weeks after injecting adalimumab (Fig. 1c). Therapeutic effect on peripheral arthritis in adalimumab-treated mice was sustained for 16 weeks after additionally injecting adalimumab 9 weeks after first injecting curdlan, while the clinical scores of arthritis remained constant in mice without adalimumab treatment.

### Increased T_H_17^+^ cell population among splenocytes of curdlan-injected SKG mice

**Figure 2:**
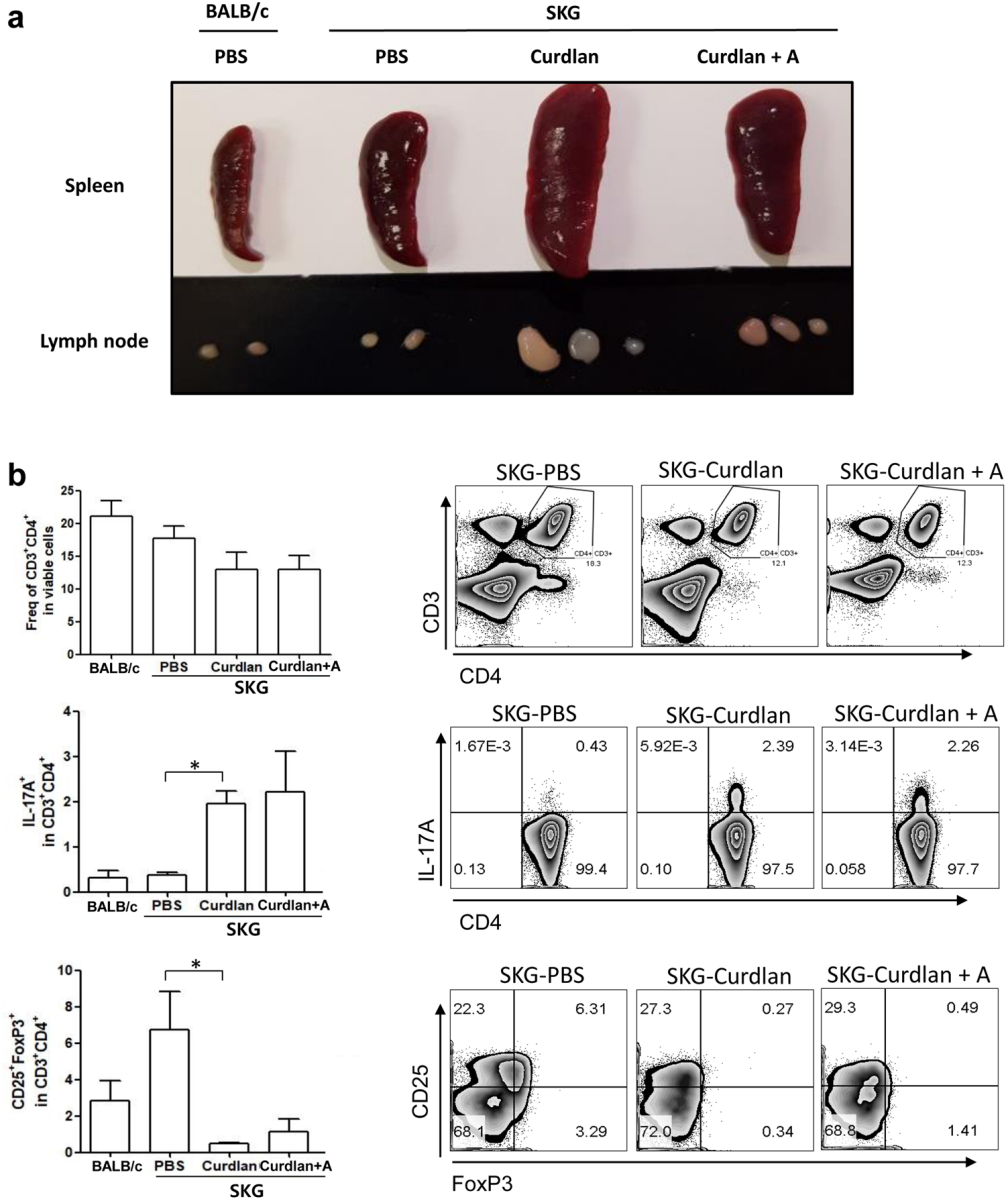
Changes in spleen and T-cell population in SKG mice following curdlan and adalimumab treatment. (**a**) Gross specimen of spleen and lymph nodes from BALB/c mice, PBS-injected SKG mice, and curdlan-injected SKG mice with or without adalimumab treatment. (**b**) flow cytometry plots showing the proportion of CD3^+^CD4^+^, IL-17A^+^, and CD25^+^FOXP3^+^ cells among splenocytes. *p < 0.05. abbreviations: curdlan + A, curdlan + adalimumab.

To evaluate changes in T-cell population in SKG mice following curdlan and adalimumab treatment, we investigated the characteristics of splenocytes in BALB/c mice, PBS-injected SKG mice, and curdlan-injected SKG mice with or without adalimumab treatment. The size of the spleen and lymph nodes was the largest in curdlan-injected SKG mice without adalimumab treatment (Fig. 2a). Flow cytometry plots showed that there were no significant differences in the frequency of CD3^+^CD4^+^ cells (CD4^+^ T cells) among the experimental groups (Fig. 2b). There was a significant increase in IL-17A^+^ cells (T_H_17 cells) and a reduction of CD25^+^FoxP3^+^ cells (T_reg_ cells) after curdlan injection. However, the populations of T_H_17 and T_reg_ cells were not significantly different between curdlan-injected SKG mice with and without adalimumab treatment (Fig. 2b).

### Adalimumab treatment reduced ^18^F-FDG uptake in PET-MRI and inflammatory cells infiltration in spinal tissue

**Figure 3:**
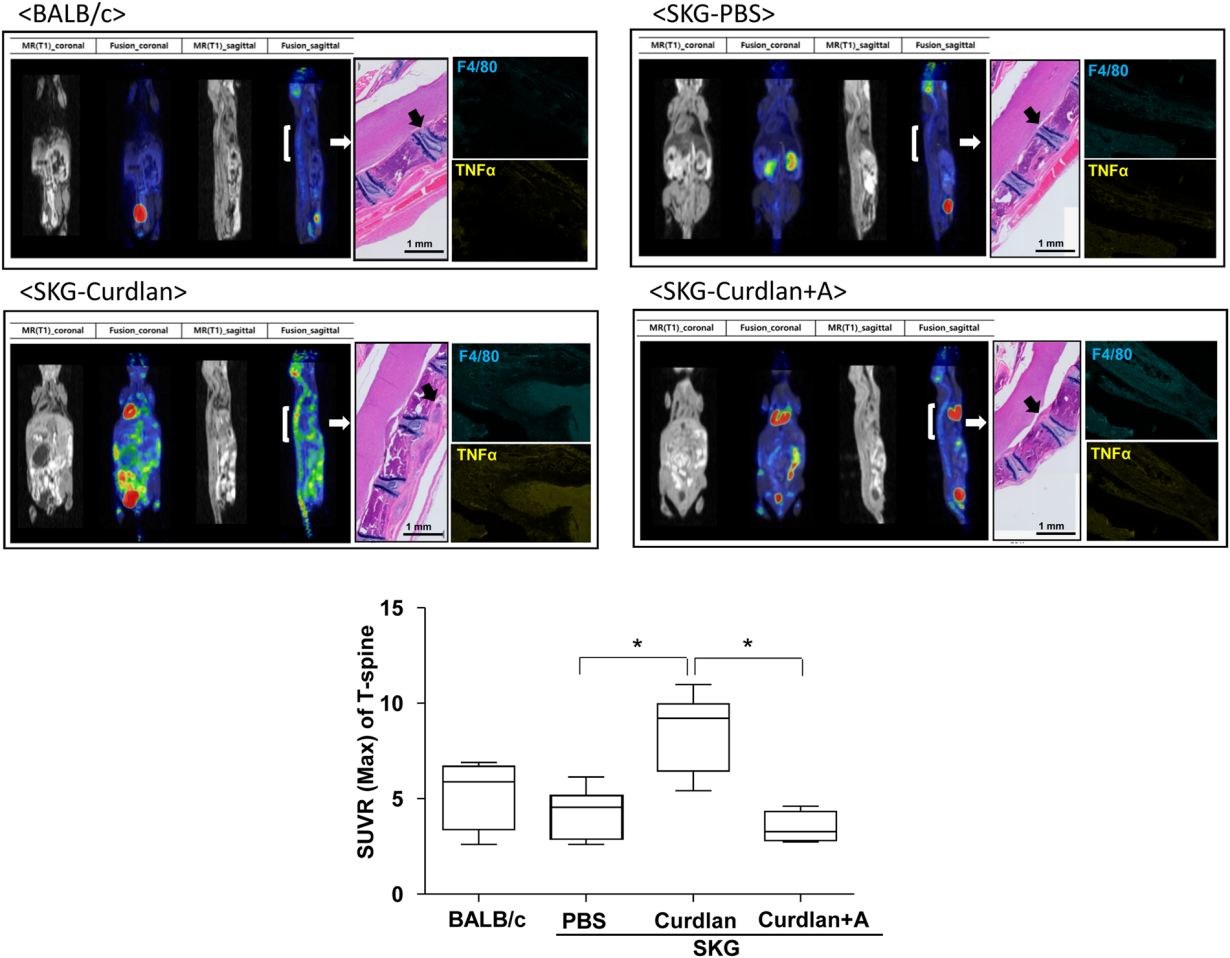
Effect of adalimumab treatment on spinal inflammation. ^18^F-FDG uptake using positron emission tomography (PET), magnetic resonance imaging (MRI), and hematoxylin and eosin staining (white arrow, ×200). Immunofluorescence staining (black arrow) images of spinal tissues. *p < 0.05. Abbreviation: curdlan + A, curdlan + adalimumab.

^18^F-FDG uptake in mice spines using PET-MRI is shown in Fig. 3. Curdlan-injected SKG mice demonstrated higher ^18^F-FDG uptake than PBS-injected SKG mice. However, the quantification of ROIs showed that the SUVR of thoracic spine was significantly reduced in adalimumab-treated mice compared with that in curdlan-injected SKG mice without treatment. These findings were further verified in histologic examination, which revealed the accumulation of inflammatory cells in the para-vertebrae of curdlan-injected SKG mice. The inflammatory lesions were not seen in adalimumab-treated mice. Further, pathogenic lesions in curdlan-injected SKG mice mainly comprised TNF-α^+^ and F4/80^+^ macrophages. These lesions were decreased in adalimumab-treated mice (Fig. 3).

### Adalimumab treatment did not decrease spinal osteoblast activity in curdlan-injected SKG mice

**Figure 4:**
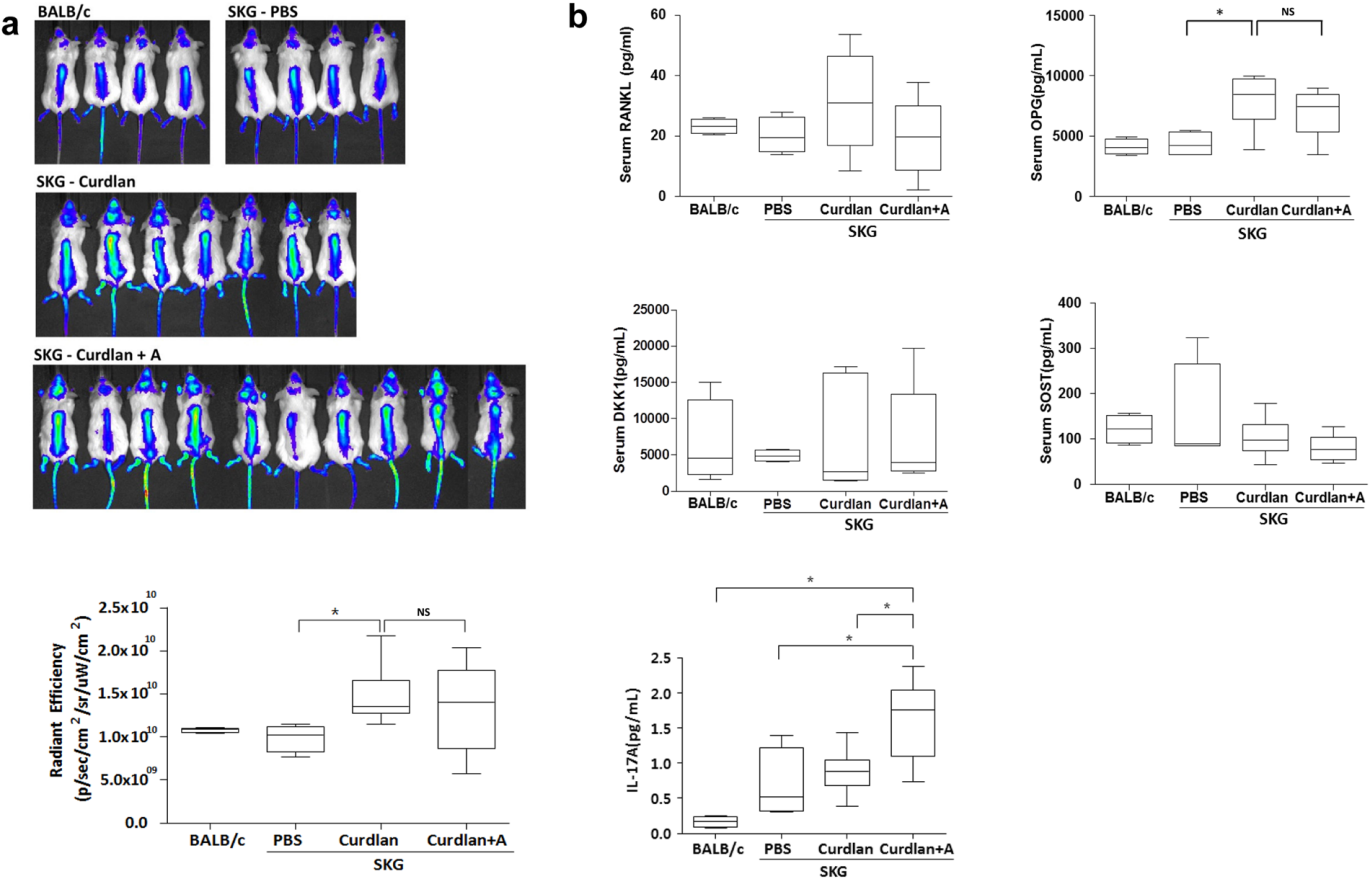
Effect of adalimumab treatment on spinal osteoblast activity. (**a**) *In vivo* imaging after injecting OsteoSense 680 EX probe and quantitatively analyzing fluorescence values. (**b**) The serum levels of bone metabolism-related cytokines and IL-17A in BALB/c mice, PBS-injected SKG mice, and curdlan-injected SKG mice with or without adalimumab treatment. *p < 0.05, **p < 0.01. Abbreviation: NS, not significant; curdlan + A, curdlan + adalimumab.

The accumulation of hydroxyapatite that suggested the osteoblast activity was assessed using a fluorescent *in vivo* bisphosphonate imaging agent (OsteoSense 680 EX). Figure 4a shows the representative biodistribution of fluorescence signals from the spines of mice. Curdlan-injected SKG mice had higher fluorescence signals than PBS-injected SKG mice, indicating a significant increase in bone formation. However, adalimumab treatment did not attenuate the osteoblast activity, which was further determined using quantitative analysis (Fig. 4a). The serum level of bone metabolism-related cytokines at the time of imaging is shown in Fig. 4b. Serum OPG level was significantly higher in curdlan-injected SKG mice than that in PBS-injected SKG mice, whereas serum RANKL level was not different, suggesting suppressed osteoclastogenic condition in curdlan-injected SKG mice. However, adalimumab treatment in curdlan-injected SKG mice did not restore the serum levels of OPG, RANKL, DKK-1, and sclerostin, supporting the results of imaging. Considering increased T_H_17^+^ cell population among splenocytes of curdlan-injected SKG mice, we additionally measured serum IL-17A levels and demonstrated increased IL-17A level in adalimumab-treated SKG mice compared to the PBS or curdlan-injected SKG mice (Fig. 4b).

## Discussion

Previous clinical studies^5–8^ have shown that TNF inhibition does not prevent spinal radiographic progression in patients with AS, while TNF inhibitors decelerated the progression of structural damages especially in early AS patients without syndesmophyte^17–21^. Additionally, an MRI study revealed that fat infiltration (post-inflammatory changes) may predict bone formation of new syndesmophytes^26^, suggesting the importance of early reduction of spinal inflammation before the potential starting point of new bone formation in AS. However, the protocols of previous clinical studies did not prohibit maintenance of nonsteroidal anti-inflammatory drugs, which are difficult to verify only the effects of TNF inhibitors. Therefore, we investigated the effect of TNF inhibition on both spinal inflammation and bone formation as soon as the peripheral arthritis was established.

In this study, we injected adalimumab in the stages of peripheral arthritis (3 weeks after first injecting curdlan), and we observed that TNF inhibition at this point reduced spinal inflammation subsequently in curdlan-injected SKG mice. In addition, inflammatory cell infiltration in the paravertebral tissue was not evident in adalimumab-treated mice. However, TNF inhibition did not decrease spinal osteoblast activity despite a reduction of inflammation in the present study. High osteoblast activity was associated with osteoproliferation and subsequent ankylosis in AS^27^. Bisphosphonate agent probe (OsteoSense 680 EX) labeled with near infrared fluorescent bisphosphonate can bind to newly-synthesized hydroxyapatite by osteoblasts^28^; thus, fluorescent intensity indicates osteoblast activity, and subsequent bone proliferation further represents the severity of AS^29^. Herein, adalimumab treatment did not decrease the osteoblast activity as assessed using fluorescent bisphosphonate imaging, despite of the effect of spinal inflammation.

OPG, which is an extracellular cytokine receptor secreted by mature osteoblasts that promotes bone formation by inhibiting osteoclast activation^30^, was not suppressed by adalimumab treatment in curdlan-injected SKG mice. Furthermore, both serum DKK-1 and sclerostin levels were not influenced by adalimumab treatment, suggesting the existence of other TNF-independent osteoproliferation pathways. In the studies, we found increased serum level of IL-17A in adalimumab-treated SKG mice. IL-17A, a cytokine having osteoclastogenic potency^31^, has been reported to accelerate osteogenesis via enhancing osteoblast differentiation from mesenchymal stem cell populations, and the subsequent activation of the osteoblasts via activation of the JAK2/STAT3 signaling pathway^32^. Walters HM *et al*.^33^ has also reported increased serum IL-17A in patients with juvenile idiopathic arthritis who were treated by etanercept, a recombinant protein of soluble TNF receptor inhibiting TNF signaling. Therefore, IL-17A pathway induced by adalimumab treatment might be supposed as one of TNF-independent osteoproliferation pathways in addition to the Wnt signaling.

T_reg_/T_H_17 imbalance is associated with the pathogenesis of autoimmune arthritis^34^. It has been reported that T_H_17 cells constitute the key arthritogenic population and are significantly higher in the blood of patients with autoimmune arthritis conditions such as RA, psoriatic arthritis, and AS^35,36^. The population of T_reg_ cells has not been fully evaluated in SpA, but several studies have shown a decrease of T_reg_ in the peripheral blood of patients with other autoimmune diseases^37–39^. In addition, previous studies have shown that TNF-α contributed to failure of T_reg_ in suppressing the proliferation of effector cells by lowering Foxp3 mRNA expression, which could be reversed by treatment with TNF-α inhibitors in autoimmune arthritis conditions such as RA^40,41^. In our study, splenocyte analysis revealed a significant increase in IL-17A^+^ cells (T_H_17) and a decrease in CD25^+^FoxP3^+^ cells (T_reg_) in curdlan-injected SKG mice compared with those in PBS-injected SKG mice. This finding is consistent with the results of previous studies^35–39^; however, adalimumab treatment did not significantly restore T_reg_/T_H_17 imbalance observed in curdlan-injected SKG mice. A possible explanation for this phenomenon is that the anti-inflammatory effect of TNF inhibitor in this model was mainly mediated by immune cells such as macrophages, mast cells, or neutrophils rather than by T cells^42,43^.

Our study has several limitations. First, the injection of adalimumab (administered during the stages of peripheral arthritis) may not have been early enough to inhibit spinal bone formation, despite the reduction of spinal inflammation. However, in an actual clinical situation, the time when arthritis is identified could be considered the earliest time to initiate treatment. Second, the mechanism underlying osteoproliferation processes was not clearly identified in this study. As mentioned above, adalimumab treatment did not decrease the osteoblast activity as observed using imaging (OsteoSense 680 EX); moreover, it did not affect bone metabolism-related cytokine levels. Further studies are warranted to clarify the mechanism of spinal bone formation in SpA. Third, the dose or treatment interval of fully humanized, anti-TNF-α monoclonal antibodies (adalimumab) may not be enough or adequate to demonstrate an inhibitory effect on TNF-α in a murine model. In this study, however, adalimumab treatment improved peripheral arthritis and spinal inflammation. Additionally, previous studies also showed anti-inflammatory effects of adalimumab in several animal models such as female BALB/c (H-2d/d) rats^44^, collagen-induced mouse arthritis model^45^, Sprague–Dawley rats^46^, and other than human transgenic animal models.

In summary, early treatment using a TNF inhibitor significantly reduced peripheral arthritis and spinal inflammation in curdlan-injected SKG mice; however, it hardly prevented spinal bone formation.

## Materials and Methods

### Mice

Female SKG mice with BALB/c background were originally obtained from Dr. S. Sakaguchi (Osaka University, Japan)^22^. Mice were maintained in the specific pathogen-free facility at Asan Institute for Life Sciences (Seoul, Korea). All animal handlings were carried out according to the protocols approved by the Institutional Animal Care and Use Committee of Asan Institute for Life Sciences (2015-14-135).

### Experimental schedule

As shown in Fig. 1a, 8-week-old, female, SKG mice were intraperitoneally injected 3-mg curdlan (Wako, Japan) suspended in 0.2-mL PBS (n = 20) or 0.2-mL PBS alone (control; n = 5) at 0 and 2 weeks. Moreover, 8-week-old, female, BALB/c mice were intraperitoneally injected 0.2-mL PBS twice (control; n = 5). All mice were monitored for up to 16 weeks. Half of the curdlan-injected SKG mice (n = 10) received adalimumab treatment (10 mg/kg of body weight) (fully humanized anti-TNF-α monoclonal antibodies, AbbVie, IL, USA)^47^ at 3 and 9 weeks after the first curdlan injection.

### Clinical scoring of peripheral arthritis

Clinical features of peripheral arthritis were monitored weekly and scored for severity on a scale of 0–4 (0 = no swelling, 1 = mild swelling and redness on the top of the foot, 2 = severe swelling and redness on the top of the foot, 3 = severe swelling and redness of the wrist or ankle joints, and 4 = severe swelling of the wrist or ankle joints and digits)^25^. The scores of affected joints were totaled.

### Splenocyte preparation

At 16 weeks after curdlan injection, spleen was filtered through a 100-μm nylon filter, and erythrocytes in filtered cells were lysed using red blood cell lysis buffer (BioLegend, CA, USA). The cells were collected by centrifugation. Dead cells were excluded from the analysis with fixable viability dye (eFluor 506; eBioscience, CA, USA). Data of flow cytometry were acquired (FACS Canto II; BD Biosciences, CA, USA) and analyzed (Flow Jo software; Tree Star, OR, USA).

### Surface and intracellular staining and flow cytometry

Anti-mouse CD16/32 (BioLegend, clone: 93) was used to block Fc receptors, and the surface markers were stained the following antibodies: FITC-conjugated anti-CD4 (BioLegend, clone: RM4-5), BV421-conjugated anti-CD3 (BioLegend, clone: 145-2C11), and APC/Cy7-conjugated anti-CD25 (BioLegend, clone: 3C7). After fix and permeabilization, cytokines and transcription factors were stained with the following antibodies: PerCP/Cy5.5-conjugated anti-RORγt (BD Biosciences, clone: Q31-378), PE/Cy7-IL-17A (BioLegend, clone: TC11-18H10.1), and Alexa Fluor 647-conjugated anti-FOXP3 (BioLegend, clone: 150D).

### Analyzing bone metabolism-related cytokines using luminex multiplex cytokine assay and single cytokine assay

Serum was obtained at 16 weeks after first injecting curdlan. Blood was allowed to clot for a minimum of 1 h at room temperature (RT) and centrifuged at 16,000 × g for 15 min at 4°C. For measuring bone metabolism-related cytokines including serum osteoprotegerin (OPG), Dickkopf-1 (DKK-1), sclerostin, and the receptor activator of nuclear factor-kappa B ligand (RANKL), the serum concentrations were determined using mouse-bone magnetic bead panel (Milliplex MAP, EMD Millipore Corporation, MA, USA.). Serum diluted with assay buffer (1:1) was mixed with antibody-linked polystyrene beads in 96-well filter-bottom plates and incubated overnight at 4 °C. After washing, the plate was incubated with mouse-bone detection antibody for 1 h at RT. Without aspiration, streptavidin–phycoerythrin was added and incubated for 30 min at RT. Subsequently, the plate was additionally washed twice and was resuspended in sheath fluid. All samples were measured in duplicates with standards (7-point dilutions) and a buffer control. Plate was read using the luminex system (Bio-plex 200; Bio-Rad Corp., CA, USA) for quantitative analysis.

For all groups, IL-17A was quantified from serum of mice using Simoa HD-1 Analyzer (Quanterix, MA, USA) at prismCDX Co., Ltd. (Gyeonggi-do, Korea). Limit of detection (LOD) for IL-17A was 0.0110 pg/mL when compensated for an eight-fold sample dilution. LOD was determined by mean blank signal + 2.5 SD.

### Spine histology and Immunofluorescence staining

At 16 weeks after curdlan injection, spines from control and treated mice were fixed in 10% buffered formalin and embedded in paraffin. Spine tissues were decalcified using ethylenediaminetetraacetic acid. Spine sections (4 μm) were cut and stained with hematoxylin and eosin.

Immunofluorescences staining was carried out as previously described^48^. Briefly, using the Opal method (Perkin Elmer, MA, USA), primary antibodies were sequentially applied to each slide. The slides were deparaffinized in xylene and rehydrated in ethanol. Antigen retrieval was performed in citrate buffer (pH 6.0) using microwave treatment. Slides were then incubated with primary rabbit antibodies for TNF-α (1:500) for 1 h in a humidified chamber at RT, followed by detection using the reagent Opal Polymer HRP Ms + Rb. TNF-α was visualized using Opal 540 (1:100). The slide was again placed in citrate buffer (pH 6.0) followed by microwave treatment and then incubated with primary rabbit antibodies for F4/80 (1:500) for 1 h in a humidified chamber at RT. This was followed by detection using the reagent Opal Polymer HRP Ms + Rb. F4/80 was then visualized using Opal 620 (1:100), and the slide was placed in citrate buffer (pH 6.0) for microwave treatment. Stained slides were scanned using the multispectral Vectra scanner and quantitative imaging system (Perkin Elmer).

### Positron emission tomography–magnetic resonance imaging (PET-MRI)

At week 15 after curdlan injection, whole-body sequential PET-MRI of mice was performed (nanoScan PET-MRI; Mediso Ltd, Hungary) (Fig. 1a). The radioligand fluorine 18-fluorodeoxyglucose (^18^F-FDG; 0.2 mCi/kg) was injected via the tail vein after a fasting period of at least 12 h. Scanning was initiated at 40 min after injecting the radioligand. After 20 min of MRI, PET was performed for 10 min. According to the previously described method^48^, contiguous axial slices (1 mm) were obtained from the whole body. Scanning parameters were as follows: repetition time = 25 s, effective echo time = 3.4 ms, field of view = 64 mm, number of excitations = 1, frequency = 128, and phase = 128. Dynamic data acquisition of PET scans was performed from 60 to 70 min after injecting ^18^F-FDG. Acquired PET images were reconstructed using the three-dimensional full detector mode with MRI-based attenuation collection with an energy level of 250–750 keV and 0.5-mm voxel size. Three-dimensional regions of interest (ROIs) were drawn over the joints using a threshold of 70%–100% of the maximum intensity, and the average signal level in the ROIs was measured by a technician. Image counts/pixels were converted to radioactivity concentrations, which were then corrected for the injected radioactivity. Results were expressed as standardized uptake value ratio (SUVR).

### Imaging with fluorescent *in vivo* bisphosphonate agent

At 16 weeks after curdlan injection, whole-body imaging of mice using fluorescent *in vivo* bisphosphonate agent was performed (OsteoSense 680 EX; Mediso Ltd, Hungary). Via the tail vein, mice were injected with the bisphosphonate agent OsteoSense 680 EX (Perkin Elmer) at a dose of 2 nmol/100 μL PBS per mouse. Back hair was removed on the same day. After 24 h, fluorescent images were obtained using an IVIS spectrum system (Perkin Elmer) with an excitation and emission wavelengths of 675 and 720, respectively, under inhalation anesthesia (Forane). After acquisition, images were spectrally unmixed (Living Image software; Caliper Life Sciences, MA, USA). ROIs with the same area were placed and measured as the mean radiant efficiency.

### Statistical analysis

All statistical analyses were performed using GraphPad Prism 5 (GraphPad Software, La Jolla, CA, USA). For comparisons between two groups, the Mann–Whitney *U* test was performed. For comparison among multiple groups, one way ANOVA with post-hoc analysis was performed. Results were presented as mean ± standard error of the mean (SEM). Error bars indicate SEM. P values < 0.05 were considered statistically significant. *p < 0.05, **p < 0.01.
